# An Observational Study on Pain Occurrence After Root Canal Treatment: Role of Operator Experience When Using a Bioceramic Sealer

**DOI:** 10.3390/jcm14134558

**Published:** 2025-06-27

**Authors:** Mihai Merfea, Ioana Sofia Pop-Ciutrila, Mindra Eugenia Badea, Ada Gabriela Delean, Oana Cimponeriu, Razvan Corneliu Pop, Maria Peter, Iulia Clara Badea, Sanda Ileana Cimpean

**Affiliations:** 1 Faculty of Dentistry, Iuliu Hatieganu University of Medicine and Pharmacy, 400012 Cluj-Napoca, Romania; mihai.merfea@umfcluj.ro (M.M.); mebadea@umfcluj.ro (M.E.B.); ada.delean@umfcluj.ro (A.G.D.); iulia.badea@umfcluj.ro (I.C.B.); sanda.cimpean@elearn.umfcluj.ro (S.I.C.); 2 County Emergency Hospital, 400347 Cluj-Napoca, Romania; cimponeriu_oana@yahoo.com (O.C.); poprazvan1905@gmail.com (R.C.P.); med.mariapeter@gmail.com (M.P.)

**Keywords:** bioceramic, calcium silicate, root canal sealer, postgraduate, specialists, root canal obturation

## Abstract

**Background and objectives**: Post-operative pain (POP) is a common complication after root canal treatment and is influenced by various clinical and patient-related factors. The present study evaluated the incidence and intensity of POP following root canal treatment using a bioceramic sealer performed by operators with different levels of experience. **Methods**: A total of 115 patients were included in this prospective observational study. Patients were treated by operators with different levels of experience: postgraduate students (PGSs) and endodontic specialists (ESs). Standardized protocols were used in two distinct appointments: an instrumentation visit and an obturation visit. Obturation was performed using the continuous wave condensation technique and Total Fill Hi-Flow BC Sealer (TFHF). POP, mastication discomfort, and sleep disturbance were assessed at 24, 48, and 72 h after the instrumentation and obturation phases using a numeric rating scale (NRS). **Results**: The results indicate significantly higher POP after the instrumentation phase, compared to the obturation phase (*p* < 0.001). The pain intensity progressively decreased over time for both phases. No significant differences were observed between the PGS and ES groups regarding POP, mastication discomfort, or sleep disturbance at any time. Sealer extrusion did not significantly impact POP (*p* > 0.05). **Conclusions**: This study found that operator experience does not significantly influence POP when a standardized protocol is followed. The use of TFHF with the continuous wave condensation technique was associated with minimal POP.

## 1. Introduction

Root canal treatment is a procedure aimed at retaining the tooth through the treatment of dental pulp and peri-radicular diseases [1]. Endodontic pathologies are recognized to have a prevalence comparable to that of other dental disorders. Therefore, endodontology postgraduate training is needed for clinicians to provide high-quality care for patients undergoing endodontic therapy [2].

Post-operative pain (POP) is an unpleasant but frequent complication after root canal therapy. A systematic review has shown that POP has a prevalence of 3 to 58% in the investigated cases [3], while a clinical trial reported up to 19% of patients experiencing severe pain [4]. The anticipation and handling of POP is an essential part of endodontic treatment, as it may be a stressful factor for both the patient and operator and may require an unscheduled emergency appointment [3]. When pain occurs following treatment, it may hinder the trust of the patient toward the operator, even if it does not influence the long-term outcome [5]. A randomized clinical trial reported that sometimes POP can exceed the pre-operative level of pain [6]. This may be due to the progress of the inflammatory process due to apical instrumentation [7], as well as chemical or microbial injuries of periapical tissues [8]. Patient-related factors like initial diagnosis [9,10,11] or tooth type [4,12] and treatment-related factors like the number of visits [13,14,15,16], instrumentation or obturation techniques [17,18,19,20], or obturation material [21,22] are linked to POP. The influence of the obturation on POP can be clarified and evaluated by splitting the treatment into two visits and differentiating it from the POP associated with the pre-operative status [10] and the mechanical preparation of the root canal [23].

Among root canal obturation techniques, warm vertical compaction using the continuous wave of condensation (CWC) technique is generally preferred by endodontists [24]. CWC softens and alters the gutta-percha phase, allowing a better fit to the root canal wall with fewer voids, and fewer radiographic translucencies [24,25,26].

The use of bioceramic sealers has become popular among operators due to their advantages such as bioactivity-enhancing periapical healing and biocompatibility [27]. A systematic review concluded that bioceramic sealers have good biological and physico-chemical properties, when compared to conventional sealers [28], with a retrospective analysis reporting an overall success rate of 90.9% [29]. Calcium silicate-based sealers were primarily developed for cold obturation techniques [30]. Consequently, Total Fill Hi-Flow BC Sealer (TFHF)—a pre-mixed and pre-loaded injectable sealer—was developed to allow the use of warm obturation techniques alongside bioceramic sealers [31], with better in vitro results compared to Total Fill BC Sealer [32].

A meta-analysis of nine randomized clinical trials showed that post-operative pain (POP) was significantly lower after root canal obturation with calcium silicate-based sealers, compared to resin-based sealers [33]; however, when compared directly, studies showed no difference between the two sealer types [34,35].

While several studies have analyzed POP after obturation using bioceramic sealers designed for cold obturation techniques [36,37], to the best of our knowledge, no study has investigated the pain after continuous wave condensation obturation along with TFHF. The operator’s experience has been shown to influence the POP after root canal treatment [4,21,38], but no study has analyzed the influence of the operator’s experience on POP after root canal obturation with bioceramic sealers.

This study aimed to evaluate and compare the incidence and intensity of POP after root canal treatment performed by two groups of operators with different experience levels using THFH and CWC.

The tested research hypotheses will be as follows:

- There is no difference in the incidence and degree of POP after the instrumentation and obturation phases of root canal treatments performed by postgraduate students and endodontic specialists;
- Pulpal and periapical diagnosis, case difficulty, and sealer extrusion have no significant effects on POP.

## 2. Materials and Methods

### 2.1. Patient Selection

This prospective observational clinical cohort study was approved by the Ethics Committee of the University of Medicine and Pharmacy “Iuliu Hatieganu” Cluj-Napoca, Romania, under approval number AVZ38 from 12 March 2024. The “Strengthening the Reporting of Observational Studies in Epidemiology” (STROBE) statement was used in preparing the study report (Table A1) [39]. All participants signed a written informed consent form prior to inclusion in the study. The consent process was conducted by a member of the research team, who provided participants with detailed information regarding the purpose of this study, the procedures involved, potential risks and benefits, data confidentiality, and the voluntary nature of participation, including their right to withdraw at any time without any consequences to their medical care. All methods were performed in accordance with the Declaration of Helsinki.

Patients referred for endodontic treatment to the Endodontics Department of the University of Medicine and Pharmacy of Cluj-Napoca within a period of 12 months were selected for this prospective study. Before treatment, the medical and dental history of the patients was recorded. Gender, age, tooth number, periapical condition, pulp diagnosis, occlusion, and proximal contacts were registered. The exclusion criteria included immunosuppressed patients, patients under 18 or over 75 years old, pregnancy, intake of analgesics 12 h prior to the treatment or consumption of antibiotic medication, teeth with previous root canal treatments, external or internal resorptions, open apexes, and root canals that made it impossible to negotiate to the apical constriction. The required sample size was calculated using G*Power 3.1 software (Kiel, Germany) for a statistical power of 0.8, with a significance threshold of 0.05 and a 0.3 effect size.

**Figure 1:**
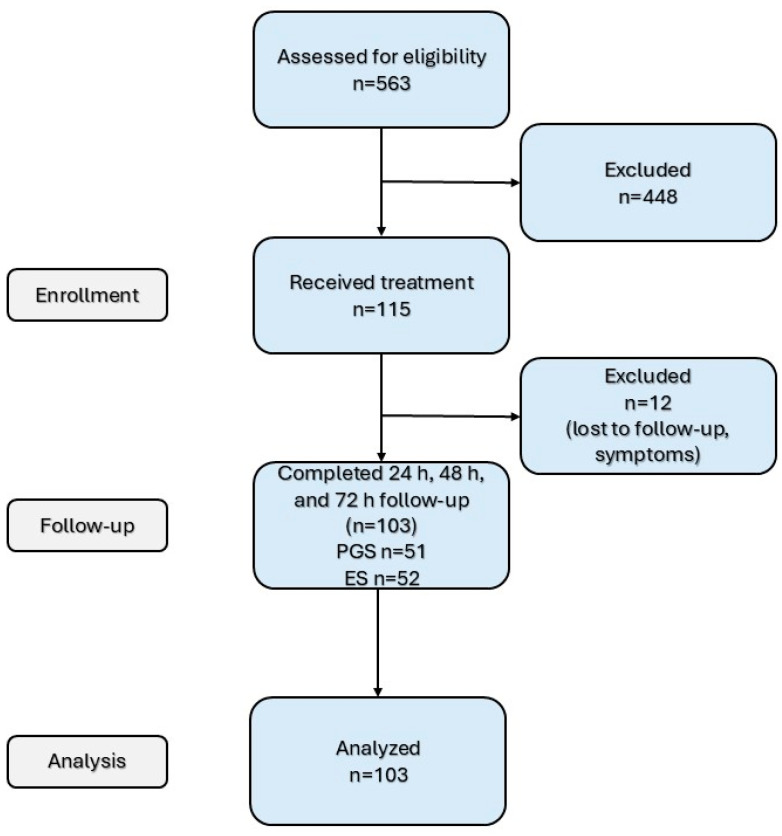
Flow chart of patient enrollment.

A total of 115 patients were considered eligible for this study (Figure 1). The patients were treated (as they presented or were referred to) by three endodontic specialists (ES) with at least 5 years of experience in this field or by three postgraduate students (PGSs) enrolled in the endodontics residency program. No randomization or blinding took place. A total of 59 patients were treated by ESs, while PGSs treated 56 patients. The treatments were performed in two visits: the instrumentation phase and the obturation phase. This protocol (i.e., in which patients require two visits) is commonly used in the endodontic department where the study took place, and it was used for all patients. Before the beginning of the study, a detailed presentation including the study protocol was held for calibration, in which all operators participated. The session included practical training using extracted teeth to ensure uniformity in treatment and obturation procedures. In cases where discrepancies arose during training, they were discussed and resolved through consensus under the supervision of an ES to ensure consistency and reliability. All of the PGSs were supervised during clinical procedures by an operator accustomed to the protocol, but the operator did not interfere with the treatment.

### 2.2. First Visit—Instrumentation Phase

**Figure A1:**
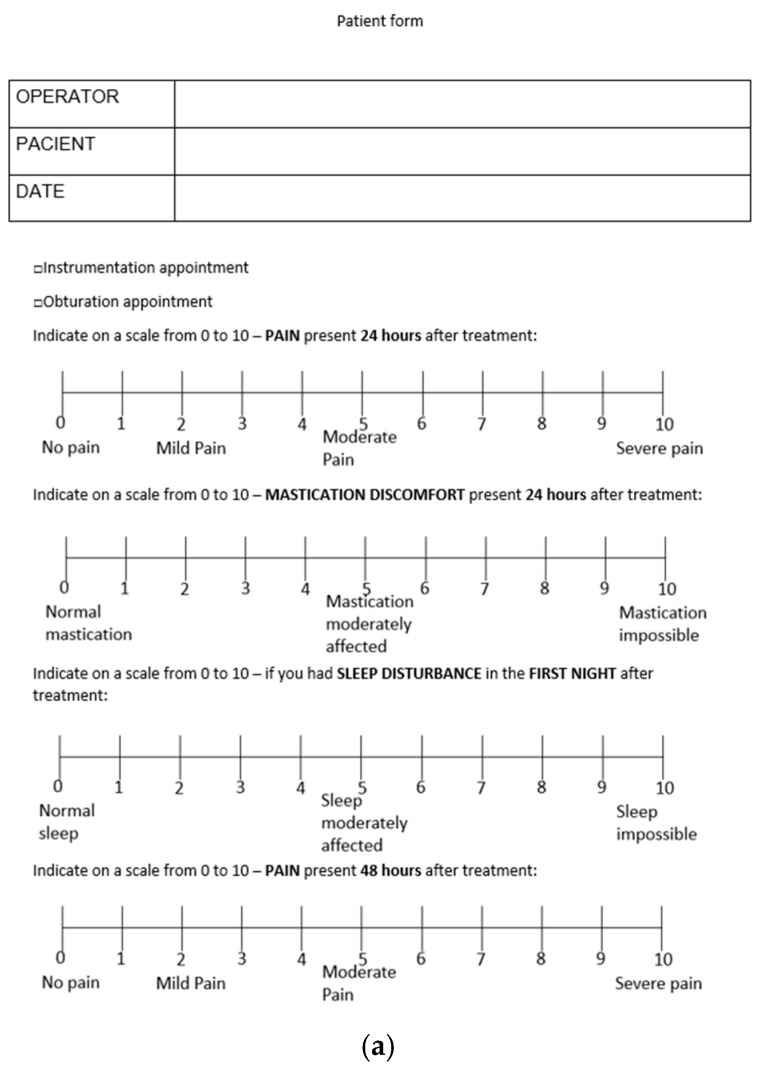

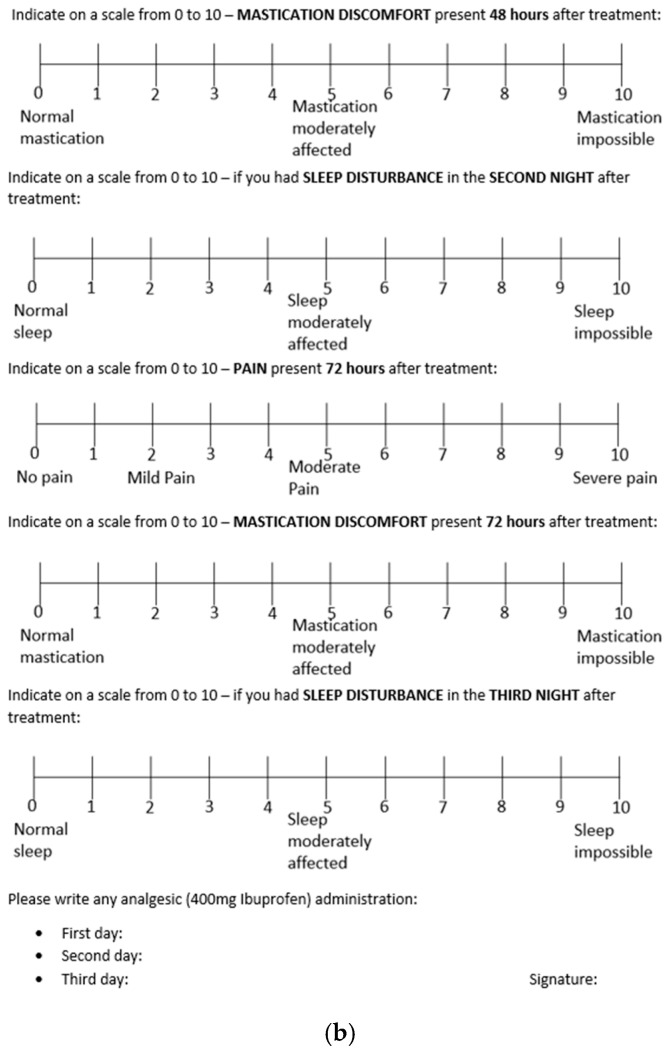
(**a**) POP patient assessment form—page 1. (**b**) POP patient assessment form—page 2.

In the first visit, each patient underwent a periapical radiograph examination (Planmeca, Helsinki, Finland), which was analyzed by one of the ESs taking part in this study, and teeth were classified as having lesions of endodontic origin (LEO) when a loss of lamina dura and periodontal ligament enlargement bigger than 2 mm was present [6]. The pulpal status was assessed using the cold test (Cerkamed, Stalowa Wola, Poland) and classified as healthy pulp, reversible pulpitis, symptomatic irreversible pulpitis, asymptomatic irreversible pulpitis, and pulp necrosis [40]. Teeth with reversible pulpitis were excluded from this study. The vitality of the tooth was confirmed clinically when entering the pulp chamber, while for asymptomatic irreversible pulpitis, deep caries involving pulp exposure was present, without any degree of symptomatology, and prolonged pulp bleeding (more than 2–3 min) [36]. The presence or absence of pain was recorded, and the peri-radicular status was checked using percussion and palpation. The case difficulty was assessed using the American Association of Endodontists (AAE) endodontic case difficulty assessment form [41]. After anesthesia with articaine containing 2% adrenaline (Septodont, Saint Maur des Fosses, France) and rubber dam isolation, the access cavity was prepared using sterile round burs (Edenta, St. Gallen, Switzerland) and Endo-Z burs (Dentsply, Konstanz, Germany). The working length was determined with a 10 K-File (VDW, Munich, Germany) and an apex locator (Root ZX, Morita, Japan), using the middle green line on the display. The Pro Taper Ultimate (Dentsply, Konstanz, Germany) rotary system was used to prepare the root canals mechanically, following the manufacturer’s instructions. Root canal irrigation consisted of 2 mL NaOCl 5.25% (Cerkamed, Stalowa Wola, Poland) between each instrument with a side-vented needle (Cerkamed, Stalowa Wola, Poland) placed 2 mm shorter than the working length using the positive pressure irrigation technique [42]. Apical patency was checked with a 10 K-File entering 0.5 mm longer than the working length [43]. The root canal was dried with paper points and dressed with temporary calcium hydroxide (Cerkamed, Stalowa Wola, Poland). Then, a sterile cotton pellet was put inside the pulp chamber, and a provisional filling (Coltosol, Coltene, Altstatten, Switzerland) was placed. Each patient received a POP assessment form (Figure A1). If needed, the patients were instructed to administer 400 mg of ibuprofen and record any analgesic administration on the form.

### 2.3. Second Visit—Obturation Phase

**Figure 2:**
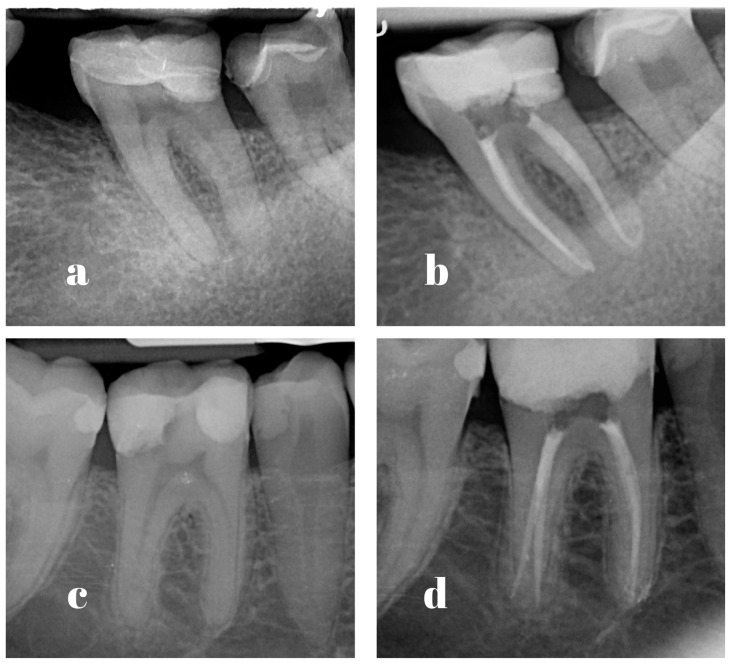
Examples of cases performed by PGSs ((**a**) pre-operative radiograph; (**b**) post-operative radiograph) and ESs ((**c**) pre-operative radiograph; (**d**) post-operative radiograph).

In the second visit, which took place 5 to 14 days after the first one, the current symptomatology of patients was assessed, and those with any degree of symptomatology at the second visit were excluded from the study. Anesthesia was again obtained using articaine containing 2% adrenaline (Septodont, Saint Maur des Fosses, France), and rubber dam isolation was performed. The provisional coronal filling was removed using an ultrasound scaler (Woodpecker, Guilin, China). Root canals were irrigated with 2 mL NaOCl (Cerkamed, Stalowa Wola, Poland), and the working length was reconfirmed using an electronic apex locator (Root ZX, Morita, Japan) and a 15 K-File (VDW, Munich, Germany). The apical diameter was measured with Ni-Ti K Files (Dentsply, Konstanz, Germany) using the following method: the Ni-Ti K File corresponding in size to the last Pro Taper Ultimate rotary file used was tried at the working length; if no resistance and binding sensation was found, the next Ni-Ti K File in size was used until a binding sensation was present [44]. The obturation technique used was the continuous wave condensation (CWC) technique, as described by Buchanan [45]. The corresponding Pro Taper Ultimate gutta-percha cone (Dentsply, Konstanz, Germany) was selected as a master cone and checked for tug-back at the working length, and then 0.5 mm was cut from the tip. Final irrigation consisted of 5 mL EDTA 17% (Cerkamed, Stalowa Wola, Poland), followed by 5 mL NaOCl (Cerkamed, Stalowa Wola, Poland). The root canal was dried using paper cones, and Total Fill BC Hi-Flow (FKG Dentaire, La Chaux-de-Fonds, Switzerland) sealer was applied inside the canal using the master cone. The gutta-percha cone was cut at the level of the root canal orifice using an electrically activated heated plugger (Kerr, Kloten, Switzerland) and condensed with a stainless-steel plugger (Kerr, Kloten, Switzerland). Next, the heat plugger was used to condense the master cone up to 4 to 6 mm shorter than the working length, followed by a pre-fitted stainless steel plugger. Next, backfill was performed using a gutta-percha injection gun (Kerr, Kloten, Switzerland). A provisional filling (Coltosol, Coltene, Altstatten, Switzerland) was placed, and a periapical radiograph was used to check the quality of the root canal (Figure 2). A blinded operator with at least 5 years of experience, who did not participate in patient treatment, assessed the radiographs and classified the outcome as short (>1 mm), adequate (<1 mm), correct, or over-obturation. The patient received a POP assessment form. If needed, the patients were instructed to administer 400 mg of ibuprofen and record any analgesic administration on the form.

### 2.4. Post-Operative Pain Assessment

Each patient received two forms (Figure A1) for POP assessment: one after the preparation phase and one after the obturation phase. Patients were instructed to mark the pain intensity on a numeric rating scale (NRS) on a horizontal line containing values from 0 to 10 [46]. The values were described as follows: 0, no pain; 1–3, mild pain; 4–6, moderate pain; 7–10, severe pain [46]. All values equal to or higher than 1 were considered as POP presence. Along with the pain intensity, the patient was asked to mark on an NRS the discomfort related to mastication and any degree of sleep disturbance caused by pain. These three indicators were assessed at 24, 48, and 72 h after the treatment [47].

### 2.5. Statistical Analysis

One-way repeated analysis of variance (ANOVA) for independent samples was conducted to evaluate differences among groups regarding continuous variables. Tukey and Bonferroni post hoc tests were performed for pairwise comparisons between groups. In the case of categorical variables, the statistical difference was evaluated via the chi-squared test. For all analyses, the threshold for statistical significance was set at *p* < 0.05. All graphical representations and analyses were performed using JASP software (JASP Team 2024, JASP version 0.19.0).

## 3. Results

A total of 103 selected patients were eligible for this study, and 12 patients were eliminated due to symptoms requiring an extra appointment, missed appointments, or failure to return pain assessment forms. The data belonging to excluded patients were removed from the analysis. Table 1 presents the demographic aspects of the two groups.

A total of 33 (32.3%) patients reported no POP after 24 h from the instrumentation visit for the whole cohort, while 70 (67.6%) complained of POP. Following the obturation phase, 53 (51.9%) patients reported the absence of POP, while the rest experienced POP (Table 2). Regarding preoperative symptoms, 43 (41.7%) patients had pain before the instrumentation visit, while all patients included in the analysis reported the absence of pain before the obturation. POP following the instrumentation visit was significantly higher (*p* < 0.01, mean difference = 1.32) when preoperative pain was present.

**Figure 3:**
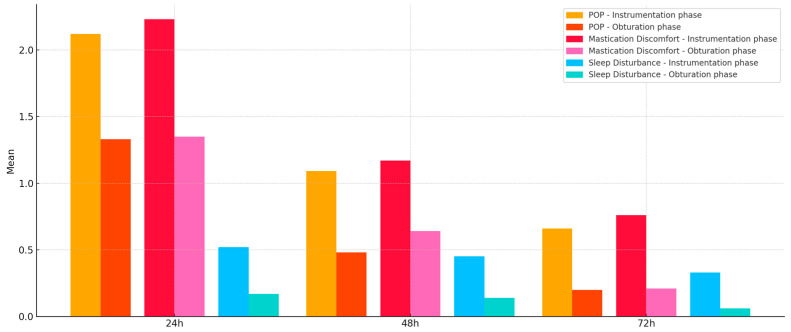
Mean values for POP, mastication discomfort, and sleep disturbance evaluation at 24 h, 48 h, and 72 h after the instrumentation and obturation phases for the whole cohort.

POP, mastication discomfort, and sleep disturbance decreased from 24 to 72 h for the whole cohort (Figure 3); thus, the results of Tukey and Bonferroni post hoc tests for the whole cohort showed that the POP, mastication discomfort, and sleep disturbance were significantly higher (*p* < 0.001) after the instrumentation phase than after the obturation one. When evaluating POP during the three days, it was observed that it progressively diminished from 24 h to 48 h (*p* < 0.001) and from 48 h to 72 h (*p* = 0.02) after the instrumentation phase. The same was noticed for the obturation phase, with significant differences for 24 h POP levels compared to those registered at 48 h (*p* < 0.001) and for 48 h compared to 72 h (*p* < 0.01). Mastication discomfort gradually decreased in both preparation and obturation phases from 24 h to 48 h (*p* < 0.001) and from 48 h to 72 h (*p* = 0.02 for the first appointment and *p* < 0.001 for the second one; Table 3). Significant differences were found between the two appointments for POP and mastication discomfort at 24 h (*p* < 0.001), 48 h, and 72 h (*p* < 0.01). The same was observed for sleep disturbance at 24 h (*p* < 0.01), 48 h, and 72 h (*p* < 0.05).

**Figure 4:**
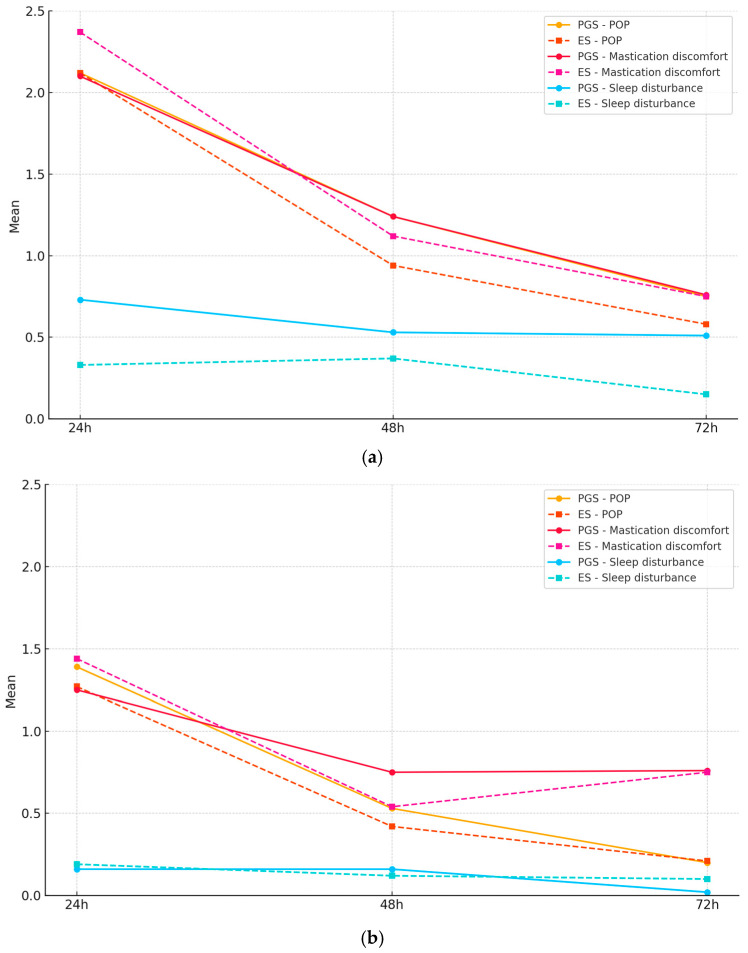
(**a**): POP, mastication discomfort, and sleep disturbance for PGSs and ESs at 24, 48, and 72 h following the instrumentation phase. (**b**): POP, mastication discomfort, and sleep disturbance for PGSs and ESs at 24, 48, and 72 h following the obturation phase.

POP, mastication discomfort, and sleep disturbance at 24, 48, and 72 h for the PGS and ES groups are presented in Figure 4a,b. The comparison between the PGS and ES groups regarding the instrumentation or obturation phases showed no significant differences for either POP, mastication discomfort, or sleep disturbance for the three days (*p* > 0.05). The same was observed when comparing results at 24, 48, and 72 h (*p* > 0.05).

Within groups, a significant difference was observed in the ES category at 24 h between the instrumentation and obturation phases for POP and mastication discomfort (*p* < 0.01), with the preparation phase having higher values. No difference was found for sleep disturbance. Within the PGS group, all three parameters (POP, mastication discomfort, and sleep disturbance) were significantly (*p* < 0.05) higher after the instrumentation appointment.

Regarding pre-operative percussion status, a positive response induced a significantly higher POP for all three days following the instrumentation visit (*p* < 0.001). However, when considering the obturation phase, the percussion test showed no statistical difference for POP (*p* = 0.67).

No significant differences were found for the two phases regarding the influence of the presence of LEO bigger or smaller than 2 mm on POP, mastication discomfort, or sleep disturbance (*p* > 0.05). The same was observed for the pulpal status, except when symptomatic and asymptomatic reversible pulpitis were compared (*p* = 0.05), with symptomatic reversible pulpitis exhibiting greater POP (mean difference = 1.89).

Regarding sealer extrusion, no significant differences were found after the obturation phase (*p* = 0.33), regardless of the operator’s experience. Sealer extrusion incidence can be seen in Table 1 and was limited in all cases to the area close to the apical foramen. At the same time, gutta-percha over-obturation happened in 6 cases, all 1 mm or less, while a short obturation was reported only in 1 case. No significant differences (*p* > 0.05) in POP were observed at 24 h, 48 h, and 72 h after the obturation appointment, regardless of the obturation quality.

Concerning the AAE difficulty, regarding the obturation visit, a moderate difficulty revealed a significantly higher POP (*p* < 0.01) compared to minimum difficulty for the whole cohort. No differences between high and moderate (*p* = 0.069) or high and minimum (*p* = 0.499) difficulty were found. AAE difficulty did not influence the instrumentation visit (*p* > 0.05). When the PGS and ES groups were compared, no differences (*p* > 0.05) were found regarding POP, mastication discomfort, or sleep disturbance regarding the influence of the case difficulty in either of the two separate visits.

No differences between tooth types (i.e., anterior, premolar, molar) regarding POP, mastication discomfort, or sleep disturbance were found (*p* > 0.05).

Regarding analgesics, 20 patients used one dose of 400 mg ibuprofen after the instrumentation phase, and 10 after the obturation visit. POP was significantly higher (*p* < 0.001) for patients who took analgesics in both phases. Data regarding the influence on the POP evolution from 24 h to 48 h can be seen in Table 4.

## 4. Discussion

Post-operative discomfort is a potentially distressing factor in endodontics, which sometimes exceeds the pre-treatment level of pain [33]. Our findings contribute to understanding factors influencing POP following endodontic treatment, particularly concerning the influence of operator experience and the choice of obturation materials. No significant differences were observed between the ES and PGS groups regarding the incidence and degree of POP after root canal treatment. Therefore, the first research hypothesis was accepted.

The POP level experienced after root canal treatment has been shown to be affected by a combination of treatment-dependent factors (ability of eliminating the infection/inflammation and of reducing pressure and swelling), including the type of rotary file, shaping techniques, irrigation solutions, and obturation technique used, as well as patient-dependent variables such as age, sex, general health condition (immune response, ability of repairing and regenerating the periapical tissues), pulpal and periapical diagnosis, pre-operative discomfort, and tooth anatomy [19,48].

Several clinical trials have studied POP following obturation with bioceramic sealers reporting similar pain values to those obtained in this study, despite differences in instrumentation and obturation protocols [21,35,49]. Meanwhile, Tan et al. reported a higher percentage of patients with no POP and fewer patients with mild or very mild POP, in contrast to the present study [34]. The reason might be the different POP assessment forms and the use of a single cone technique in Tan et al.’s research.

Endodontic treatment performed in multiple visits, as it was done in the present study, is common and allows for thorough cleaning of complex root anatomies [50]. Two systematic reviews have concluded that it may lead to lower immediate POP and flare-ups [13,15], though several studies have reported no significant differences compared to single-visit treatments [14,16,51]. In the present study, the period between the two phases was between 5 and 14 days. This is in accordance with the literature, which states that POP decreases significantly 4 days following the instrumentation appointment [23], and most of the flare-ups emerge in the first 3 days [52]. The second appointment can be postponed up to two months [53]. Instrumentation can cause bacterial extrusion and apical injuries, leading to inflammation and POP [54]. Different clinical trials have reported that mechanical glidepath [6] and 1.3% NaOCl irrigation could reduce POP [55], while rotary instrumentation was shown in a meta-analysis to exhibit less debris extrusion and lower POP [56]. Pro Taper Ultimate, the rotary system used, was reported to extrude less debris when compared to other continuous rotation systems like Pro Taper Gold [57], and similar results were noted compared to TruNatomy [58]. A possible explanation could be the different design of the Pro Taper Ultimate files, which enhances the removal of the debris in a coronal direction. In the present study, all operators used the same standardized protocol that was typically employed in their dental hospital, which they had agreed upon and practiced before patient enrollment. The multitude of instruments, techniques, and irrigation protocols available makes it difficult to evaluate and compare their effect on POP in a controlled environment.

Consistent with prior studies, patients presenting with preoperative pain tend to experience greater POP after the first appointment. Glennon et al. observed in a multi-visit treatment that preoperative pain almost tripled the odds of POP [59], while another clinical trial reported a doubling of the percentage of patients presenting POP when pain was present at the beginning of the treatment [60].

The pulpal and periapical diagnosis has been demonstrated in other studies to affect post-treatment pain following the endodontic procedure [9,48]. In our study, only positive percussion—an important procedure for deciding the diagnosis—and symptomatic reversible pulpitis significantly influenced POP for the instrumentation visit (*p* < 0.001 and *p* = 0.05, respectively). Therefore, the second research hypothesis was partially accepted. Tenderness to percussion tests before treatment has been found by several clinical trials to influence the intensity of POP after the instrumentation visit, despite different instrumentation protocols and POP assessment forms [9,11,61]. Regarding the POP following the obturation phase, no difference between positive and negative percussion tests was reported, similar to the results reported by Jang et al. [9]. One study has reported no difference in the degree or duration of POP after the endodontic procedure between vital and non-vital teeth, despite dissimilar obturation techniques, confirming the present findings [62]. However, a prospective study using different instrumentation protocols reported that vital pulp might predict a higher POP [63]. In the present study, tooth vitality testing was performed using the cold test due to equipment limitations, even though the electric pulp test was reported to be more accurate in a systematic review, and it might be more suited for differential diagnosis between a healthy pulp and asymptomatic irreversible pulpitis [64,65]. However, the pulp status was confirmed clinically for asymptomatic irreversible pulpitis, with deep caries touching the pulp and prolonged bleeding [36]. Regarding periapical translucencies, some studies have shown that the presence of a radiolucent lesion might result in higher POP [10,66], while another study found a lower POP when the patients presented radiographic apical translucency [67]. Differences in treatment protocols, definitions of apical lesions, and POP assessment may be the causes for these discrepancies. In the present study, no differences were found when periapical lesions were present or not.

As in other studies where treatments were performed by either general dentists or endodontic specialists [4,9], tooth type (anterior, premolar, or molar) did not influence POP, even though previous findings suggested that molars treated by dentists with the primary focus on endodontics or endodontic specialists are associated with higher POP due to their complex root anatomy and increased likelihood of apical tissue disruption during instrumentation and obturation [12,48]. When AAE difficulty was compared in our research, the patients reported higher scores in cases of medium difficulty (compared to minimum difficulty) for the obturation phase, while no difference was observed for the instrumentation phase. Thus, the conflicting results may be due to different study designs and a uniform protocol applied in our study by practitioners with current or completed advanced endodontic training.

The CWC technique used for obturation in the present study was demonstrated to exhibit good dentinal tubule penetration and less void formation, particularly when used with TFHF [25]. However, another study reported that this technique could create thermal injuries in the peri-radicular tissues, produced by the temperature increase on the root surface, thus increasing the incidence of POP [68]. Similar to Ali et al.’s study, in this research, low POP levels were registered when asymptomatic teeth were obturated [20].

Regarding sealer extrusion, different studies have reported no difference in POP between Ah-Plus and Endosequence BC sealers [35,37], or between resin- and calcium silicate-based materials [69]. Although bioceramic sealers seem to exhibit more extrusion than other materials, several investigations have reported no correlation between calcium silicate-based sealer extrusion and POP [34,37,69], as observed in the present study (*p* > 0.05). In contrast, one prospective clinical trial has reported a direct correlation between sealer extrusion and POP [21]. These different results may be due to differences in study design, treatment protocol, number of visits, or obturation techniques.

When comparing the POP outcomes between the two groups of operators, no significant difference was observed between them (*p* > 0.05). The clinical implication of these findings could reflect the importance of careful supervision of less-experienced operators, which could minimize the risk of potential mistakes such as over-instrumentation and over-obturation that may lead to higher POP. This may suggest that training programs should instruct trainees to implement a consistent protocol to be used during treatment. The influence of the operator’s experience on the outcome and POP of endodontic treatments has been studied for a long time. A prospective study has reported that undergraduate students had better results in terms of flare-ups than those treated by postgraduate students or specialists, suggesting that the cause might be the longer duration spent cleaning and shaping [70]. In contrast, two studies comparing general dentists and endodontic specialists reported no difference in POP [4,59], while several studies have reported a low POP incidence and good treatment outcomes in treatments performed by postgraduate or undergraduate students [21,71].

The pain assessment in this study was conducted using a numeric rating scale (NRS)—a widely recognized and validated tool for quantifying subjective pain intensity [72]. This method has been shown to provide reliable and reproducible measurements of pain intensity across diverse clinical settings [73]. The simplicity of this scale allowed for the collection of data regarding several symptoms such as POP, mastication discomfort, and sleep disturbance. However, using the Short Form McGill Pain Questionnaire—2 that asks the patient to describe the pain (sharp, throbbing, tiring, or thermal effects like hot or cold) [74] or a form that registers more details regarding the post-operative quality of life (like chewing, speaking, carrying out daily functions, or social relations) might give more information regarding the link between these factors and POP [75]. Assessments were conducted at 24, 48, and 72 h after each treatment phase in order to capture the evolution of the pain. This timeframe aligns with established recommendations for monitoring post-operative discomfort in endodontic studies, as the peak of inflammatory response and associated pain typically occurs within the first 24–48 h [7].

Regarding pre-medication, a clinical trial has reported that the use of non-steroidal anti-inflammatory drugs (NSAIDs) prior to treatment reduced the POP following the endodontic treatment [76]. In the current study, before any of the two visits, no patient took any NSAIDs, while those administered after the treatment did not have a significant effect on pain evolution (*p* > 0.05). Antibiotic medication has been found not to affect POP in a systematic review [77]; however, we excluded any patient who took antibiotics before the first and second visits.

When interpreting the results of this study, several limitations should be considered. The single-center design of this study may lead to potential biases, such as the characteristics and inadequate blinding of patients and operators, as well as equipment quality, potentially limiting the generalizability of the findings. The non-random allocation may have affected the consistency and comparability of the outcomes. The supervisor’s feedback to PGSs may positively impact the treatment quality. A long-term follow-up would also be necessary to obtain a broader perspective on these findings. The study design focused exclusively on a single type of bioceramic sealer and a specific obturation technique (CWC), which may not fully reflect the diverse clinical scenarios encountered in routine practice. Further, the reliance on patient-reported outcomes introduces a degree of reporting bias. Additionally, factors such as individual pain thresholds, pre-operative anxiety, and variations in anatomical complexity were not fully accounted for. Finally, the study’s exclusion criteria—such as the absence of symptomatic cases at the obturation phase—may not apply to all clinical conditions.

Future research should aim to address the limitations of this study by conducting randomized clinical trials and including multi-center trials to increase the generalizability of findings. Comparative studies exploring different bioceramic sealers, obturation techniques, and instrumentation systems are needed to establish optimal strategies for POP minimization. Additionally, investigations should focus on long-term outcomes, such as periapical healing and re-treatment success, when using bioceramic sealers. These could create a benchmark for proposing clinical guidelines regarding standardized protocols for trainee programs and usual clinical practice. Advanced imaging modalities such as cone-beam computed tomography (CBCT) could enable more precise evaluations of sealer extrusion and obturation quality. Furthermore, the role of adjunctive therapies—including pre-medication with NSAIDs, corticosteroids, or other analgesics—should be explored in greater detail. Studies that integrate patient-specific factors, such as pre-operative anxiety, anatomical variations, genetic predispositions to pain sensitivity, investigate the characteristics of POP and how it affects post-operative quality of life, would enable a more comprehensive understanding of POP in the endodontic context.

## 5. Conclusions

This study provides important insights into the factors influencing POP. The findings highlight the influence of treatment protocols, materials, and patient-specific factors. Operator experience did not affect POP when standardized protocols were followed. POP was more pronounced after the instrumentation phase than the obturation phase, emphasizing the potential impact of the shaping procedure and apical tissue injuries. Positive percussion influenced POP after the instrumentation phase, but not following the obturation phase, while sealer extrusion and case difficulty had no influence on POP. The use of a bioceramic sealer designed for warm obturation techniques, such as TFHF, demonstrated minimal POP, supporting its application in modern endodontic practice.

## Figures and Tables

**Table 1:** Demographics and distribution of clinical features.

Factor		PGS	ES	Total	*p* ^a^
**Age ^1^**		42.3 ± 17.2	42.2 ± 15.3	42.3 ± 16.7	0.980
**Gender**	Female	33	34	67	0.940
	Male	18	18	36	
**Tooth type**	Anterior	15	7	22	0.100
	Premolar	10	9	19	
	Molar	26	36	62	
**AAE Difficulty**	Minimum	19	14	33	0.500
	Moderate	14	15	29	
	High	18	23	41	
**Pulp Status**	Vital (healthy)	8	8	16	0.402
	Symptomatic Irreversible Pulpitis	14	10	24	
	Asymptomatic Irreversible Pulpitis	5	9	14	
	Necrosis	25	24	49	
**Percussion**	Positive	15	20	35	0.330
	Negative	36	32	68	
**LEO**	No	34	34	68	0.700
	Leo < 2 mm	9	12	10	
	Leo > 2 mm	8	6	14	
**Extrusion**	Yes	11	20	31	0.060
	No	40	32	72	
**Obturation Quality**	Correct	39	46	85	0.280
	Adequate	8	3	11	
	Short	1	0	1	
	Overfilling	3	3	6	

^1^ Mean ± standard deviation (SD); *p*, significance between groups (PGS and ES); LEO, lesion of endodontic origin; ^a^ α < 0.05.

**Table 2:** Presence or absence of POP 24 h after the instrumentation phase or obturation phase for the two groups.

	POP	PGS	ES	Total	*p* ^1,a^
Instrumentation Phase	No	17 (34.0%)	16 (30.7%)	33 (32.3%)	0.946
	Yes	34 (66.0%)	36 (69.2%)	70 (67.6%)	
Obturation Phase	No	23 (46.0%)	30 (57.6%)	53 (51.9%)	0.279
	Yes	28 (54.0%)	22 (42.3%)	50 (48.0%)	
*p* ^2,a^		0.311	0.010		

*p*^1^, significance between groups (PGS and ES) for the two phases; *p*^2^, significance between phases (instrumentation or obturation) for each group; ^a^ α < 0.05.

**Table 3:** Mean difference and significance between different timepoints for the instrumentation and obturation phases regarding POP, mastication discomfort, and sleep disturbance.

		Instrumentation Phase	Obturation Phase
POP	24 h vs. 48 h	1.03 *	0.85 *
	24 h vs. 72 h	1.46 *	1.13 *
	48 h vs. 72 h	0.43 ***	0.27 **
Mastication discomfort	24 h vs. 48 h	1.06 *	0.71 *
	24 h vs. 72 h	1.48 *	1.14 *
	48 h vs. 72 h	0.42 ***	0.43 *
Sleep disturbance	24 h vs. 48 h	0.08	0.04
	24 h vs. 72 h	0.19	0.12
	48 h vs. 72 h	0.12	0.08

* *p* < 0.001; ** *p* < 0.01; *** *p* < 0.05.

**Table 4:** Mean difference and significance between POP at 24 h and 48 h between patients who took or not analgesics.

	Instrumentation Phase	Obturation Phase
Analgesics (MD)	1.08	0.77
No Analgesics (MD)	0.80	1.60
*p* ^a^	0.47	0.11

^a^ α < 0.05; MD—mean difference between POP at 24 h and POP at 48 h.

## Data Availability

Data available on request from the authors due to restrictions (privacy, ethics, patient data).

## Abbreviations

The following abbreviations are used in this manuscript:

AAE	American Association of Endodontics
ESs	Endodontics specialists
CWC	Continuous wave condensation
LEO	Lesion of endodontic origin
NRS	Numeric rating scale
NSAID	Non-steroidal anti-inflammatory drug
POP	Post-operative pain
PGSs	Postgraduate students
SD	Standard deviation
TFHF	Total Fill Hi-Flow BC Sealer

## Appendix A

**Table A1:** STROBE Statement Checklist—for cohort studies.

	Item No.	Recommendation	Article Section
Title and abstract	1	(a) Indicate the study’s design with a commonly used term in the title or the abstract	Abstract—material and methods
		(b) Provide in the abstract an informative and balanced summary of what was done and what was found	Abstract
Introduction			
Background/rationale	2	Explain the scientific background and rationale for the investigation being reported	2nd, 6th paragraph
Objectives	3	State specific objectives, including any prespecified hypotheses	7th, 8th paragraph
Methods			
Study design	4	Present key elements of study design early in the paper	Patient selection—1st, 3rd paragraph
Setting	5	Describe the setting, locations, and relevant dates, including periods of recruitment, exposure, follow-up, and data collection	Patient selection 2nd; 3rd paragraph; Figure 1
Participants	6	(a) Give the eligibility criteria, and the sources and methods of selection of participants; describe methods of follow-up	Patient selection—2nd paragraph; POP assessment form
		(b) For matched studies, give matching criteria and number of exposed and unexposed	n/a
Variables	7	Clearly define all outcomes, exposures, predictors, potential confounders, and effect modifiers; give diagnostic criteria, if applicable	First visit; POP assessment form
Data sources/measurement	8	For each variable of interest, give sources of data and details of methods of assessment (measurement); describe comparability of assessment methods if there is more than one group	POP assessment form
Bias	9	Describe any efforts to address potential sources of bias	Patient selection—3rd paragraph; Second visit; POP assessment form
Study size	10	Explain how the study size was arrived at	Patient selection—3rd paragraph
Quantitative variables	11	Explain how quantitative variables were handled in the analyses. If applicable, describe which groupings were chosen and why	Statistical analysis
Statistical methods	12	(a) Describe all statistical methods, including those used to control for confounding	Statistical analysis
		(b) Describe any methods used to examine subgroups and interactions	Statistical analysis
		(c) Explain how missing data were addressed	No missing data
		(d) If applicable, explain how loss to follow-up was addressed	Results—1st paragraph
		(e) Describe any sensitivity analyses	None (low N)
Results			
Participants	13	(a) Report numbers of individuals at each stage of study—e.g., numbers potentially eligible, examined for eligibility, confirmed eligible, included in the study, completing follow-up, and analyzed	Figure 1
		(b) Give reasons for non-participation at each stage	Results—1st paragraph
		(c) Consider use of a flow diagram	Figure 1
Descriptive data	14	(a) Give characteristics of study participants (e.g., demographic, clinical, social) and information on exposures and potential confounders	Table 2
		(b) Indicate number of participants with missing data for each variable of interest	Results—1st paragraph
		(c) Summarize follow-up time (e.g., average and total amount)	Figure 1
Outcome data	15	Report numbers of outcome events or summary measures over time	Results—2nd paragraph
Main results	16	(a) Give unadjusted estimates and, if applicable, confounder-adjusted estimates and their precision (e.g., 95% confidence interval); make clear which confounders were adjusted for and why they were included	No confounders/control variables.
		(b) Report category boundaries when continuous variables were categorized	Results—2nd paragraph
		(c) If relevant, consider translating estimates of relative risk into absolute risk for a meaningful time period	
Other analyses	17	Report other analyses done—e.g., analyses of subgroups and interactions, and sensitivity analyses	Patient selection—3rd paragraph Power analysis
Discussion			
Key results	18	Summarize key results with reference to study objectives	1st paragraph; 5th paragraph
Limitations	19	Discuss limitations of the study, taking into account sources of potential bias or imprecision; discuss both direction and magnitude of any potential bias	13th paragraph
Interpretation	20	Give a cautious overall interpretation of results considering objectives, limitations, multiplicity of analyses, results from similar studies, and other relevant evidence	1st paragraph; Conclusions
Generalizability	21	Discuss the generalizability (external validity) of the study results	Discussion—13th paragraph; 14th paragraph
Other information			
Funding	22	Give the source of funding and the role of the funders for the present study and, if applicable, for the original study on which the present article is based	None
